# White-Sutton syndrome and congenital heart disease: case report and literature review

**DOI:** 10.1186/s12887-023-03972-9

**Published:** 2023-04-04

**Authors:** Jing Duan, Yuanzhen Ye, Jianxiang Liao, Li Chen, Xia Zhao, Chao Liu, Jialun Wen

**Affiliations:** 1 Department of Neurology, Shenzhen Children’s Hospital, 7019# Yitian Road, Futian District, Guangdong Province 518038 Shenzhen, PR China; 2 Department of Bioinformatics, Berry Genomics Co. Ltd, Beijing, China

**Keywords:** *POGZ*, White-Sutton syndrome, Congenital heart disease, Developmental delay, Case report

## Abstract

**Background:**

White-Sutton syndrome is an autosomal dominant neurodevelopmental disorder caused by heterozygous mutation in *POGZ* (Pogo Transposable Element Derived with ZNF Domain). This syndrome is characterized by delayed psychomotor development apparent in infancy and abnormal facial features. To date, 80 cases have been reported in the literature; however, the phenotypic characterizations remain incomplete.

**Case presentation:**

We herein describe a 2-year-old girl harboring a novel frameshift de novo *POGZ* variant: c.2746del (p.Thr916ProfsTer12). This patient presented with multisystem abnormalities affecting the digestive tract and neurological functioning, as well as congenital heart disease, which involved an atrial septal defect (18 × 23 × 22 mm) with pulmonary arterial hypertension (42 mmHg). The relationship between congenital heart disease and White-Sutton syndrome as described in both the GeneReview and OMIM databases (#616,364) remains unclear. A review of the current literature revealed 18 cases of White-Sutton syndrome with *POGZ* variants and congenital heart disease, and we summarize their clinical features in this study.

**Conclusions:**

Our findings based on the present case and those in the literature indicate a relationship between *POGZ* mutation and congenital heart disease.

**Supplementary Information:**

The online version contains supplementary material available at 10.1186/s12887-023-03972-9.

## Background

*POGZ* encodes a zinc finger protein that is mainly found in the nucleus [1] and known to be involved in neuronal proliferation, chromatin remodeling, cell cycle progression and gene transcription regulation [2, 3]. Previous research has shown that *POGZ* is enriched in cerebrocortical and hippocampal neurons of early mouse embryos and regulates cortical neuronal development by promoting neuronal differentiation [4]. De novo disruptive mutations of *POGZ* are associated with White-Sutton syndrome, a syndromic neurodevelopmental disorder characterized by developmental delay, cerebral malformation, hearing loss, facial dimorphisms, and seizures [5, 6]. To date, 80 cases of White-Sutton syndrome have been reported [5–19]. However, the phenotypic characterizations of this syndrome remain incomplete.

Herein, we present a case of White-Sutton syndrome with a novel *POGZ* frameshift mutation. The patient presented multisystem manifestations, including developmental delay, hypokalemia, congenital heart disease, incomplete intestinal obstruction, and dystonia. A review of the existing literature returned 18 additional cases of White-Sutton syndrome with de novo *POGZ* variants that presented with congenital heart disease. The findings of the present case and literature analysis provide insight for further establishing the phenotypic spectrum of White-Sutton syndrome.

## Case presentation

**Fig. 1:**
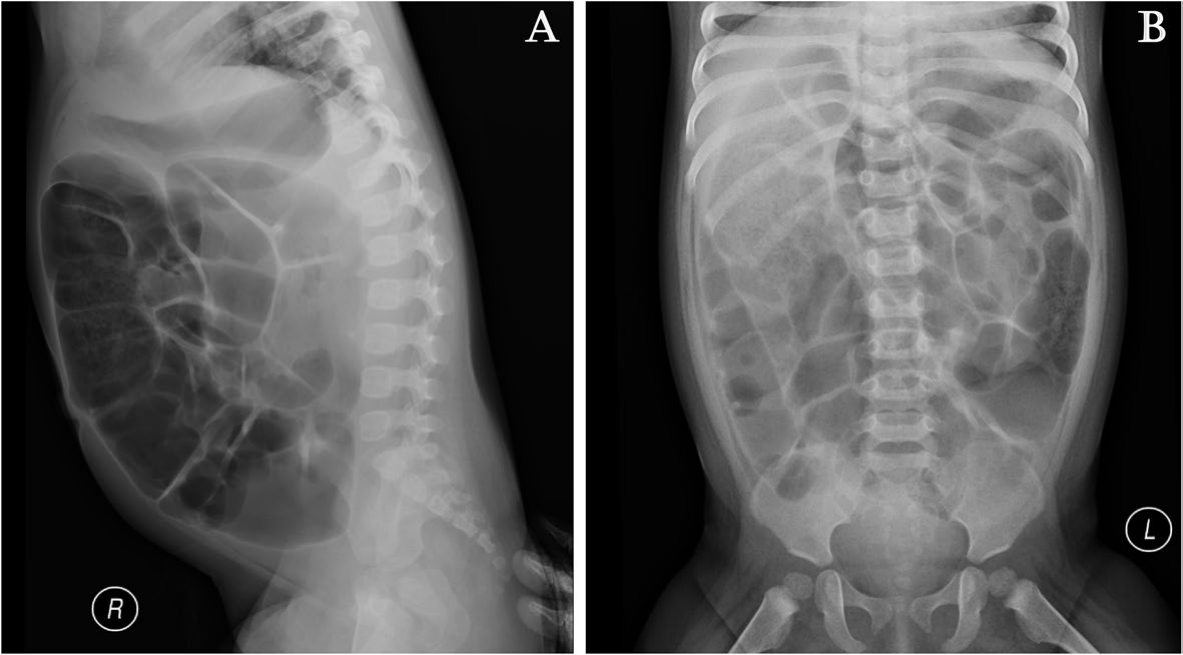
At 1 year of age, abdominal X-ray revealed mechanical ileus. **A** Right lateral abdominal radiograph; **B ** Supine abdominal radiograph

We report a case of White-Sutton syndrome in a 2-year-old girl. She was the second child of healthy and unrelated Chinese parents. She was born at 39 weeks of gestation by cesarean section, with a birth weight of 2840 g. The mother had gestational diabetes mellitus. The patient had a 20-year-old brother who was healthy, and her family history was negative for heart disease, epilepsy, and other neurological disorders. Nineteen hours after delivery, the girl was admitted to the neonatal unit due to repeated vomiting and diagnosed with digestive tract bleeding, which was managed with fasting and thrombin. The passage of meconium was not delayed, but abdominal distension was observed from 4 days after birth and persisted. Abdominal ultrasound showed a dilated bowel and bowel gas. Abdominal distension recurred several times over the next 2 years, culminating in mechanical ileus (Fig. 1) at the age of 1 year. Mechanical ileus was improved by fasting, gastrointestinal decompression, and glycerin enema.

At the age of 5 months, the patient was diagnosed with an atrial septal defect (18 × 23 × 22 mm), and pulmonary arterial hypertension (42 mmHg) was recorded for the first time. Atrial septal defect closure surgery was performed when the patient was 5 months of age, and the patient was treated with digoxin (0.1 mg/kg.d), spironolactone (2.4 mg/kg.d), and hydrochlorothiazide (0.8 mg/kg.d) after surgery. The patient had recurrent pneumonia after surgery and was admitted to the ICU 6 months after surgery for heart failure. Her left ventricular ejection fraction dropped to 22% at the lowest recording. At the 1-year follow-up after ICU discharge, the patient’s left ventricular ejection fraction ranged from 47 to 55%.

At the age of 9 months, she presented with epileptic spasm with hypsarrhythmia several times a day. She was successively treated with courses of topiramate (TPM; maximum dosage of 5 mg/kg.d), valproate (VPA; maximum dosage of 24 mg/kg.d), and cocktail therapy. No obvious seizure attack was observed between the ages of 13 and 19 months after combined treatment with TPM, VPA and cocktail therapy, which was a combination nutraceutical therapy consisting of vitamin B1 50 mg/d, vitamin B2 100 mg/d, vitamin C 200 mg/d, vitamin E 100 mg/d, L-carnitine 1000 mg/d, and coenzyme Q10 100 mg/d. At 19 months old, seizure returned, occurring several times a day, and did not improve with successively administered courses of levetiracetam (LEV; maximum dosage of 20 mg/kg.d), vigabatrin (VGB: the maximum dosage of 160 mg/kg.d), nitrazepam (NZP: the maximum dosage of 0.07 mg/kg.d) and clobazam (CLB; maximum dosage of 0.27 mg/kg.d). Adrenocorticotropic hormone (ATCH, 1.5 IU/kg) was added to the combined levetiracetam, vigabatrin and cocktail therapy for 2 weeks when the patient was 1 year and 10 months old. Thereafter, prednisone was continued before being gradually reduced and withdrawn over 1 month. The frequency of seizures decreased to two times per week.

From 1 year of age, the patient suffered from sleep disturbance, which mainly manifested as light sleep, often crying in sleep, and being difficult to soothe. The patient had several dysmorphic features, including a high-arched palate, frontal bossing, a congenital preauricular fistula, a tented mouth, a broad nasal root, a flat nasal bridge, and tongue protrusion. Brain magnetic resonance imaging performed at 1 year showed cerebral atrophy associated with enlargement of the supratentorial ventricles, thinned corpus callosum, and delayed myelination. She did not pass the newborn hearing screening conducted with otoacoustic emissions testing, and hearing loss was confirmed by otoacoustic emissions testing at the age of 1 year.

Peripheral venous blood samples were collected from the proband and her parents with their informed consent. Chromosomal microarray analysis for the proband was performed using Affymetrix Cytoscan 750 K. The results of the chromosomal microarray analysis and mitochondrial genetic testing for the proband were normal. The results of prenatal karyotype analysis on a cord blood sample also were normal. Trio-based WES revealed that the *POGZ* gene had a *de novo* heterozygous frameshift mutation [NM_015100.4:c.2746delA (p.Thr916ProfsTer12)], which was not found in current population databases (dbSNP, GnomAD, and ExAC). Most previously reported mutations in the *POGZ* gene are null variant [5, 7, 20]. According to the guidelines of the American College of Medical Genetics and Genomics (ACMG) and the Association of Molecular Pathology (AMP), the variant identified in the present case is considered pathogenic.

At the last follow-up at 2 years of age, the patient was experiencing a seizure every 3–5 days. Her parents had stopped all anti-seizure medications against medical advice, and she was receiving traditional Chinese massage. Developmentally, she could turn over, sit without support, make eye contact, and laugh, but could not stand or speak.

## Discussion and conclusions

The clinical spectrum of White-Sutton Syndrome is relatively wide, with known manifestations including autism spectrum disorder, developmental delays, and intellectual disability [5, 7, 17, 20, 21]. Additional commonly reported features include feeding and gastrointestinal problems, seizures, sleep problems, hearing loss, vision problems and genitourinary abnormalities. However, the association of congenital heart disease with *POGZ* haploinsufficiency has not been well characterized in the previous literature. As a result, the relationship between heart disease and White-Sutton syndrome as described in both the GeneReview [21] and OMIM databases (#616,364) remains unclear. The present case report describes a new patient with a pathogenic variant of the *POGZ* gene who presented with congenital heart disease. This case was then compared to all cases of patients with *POGZ* mutations and heart disease that were found in the literature.

**Fig. 2:**
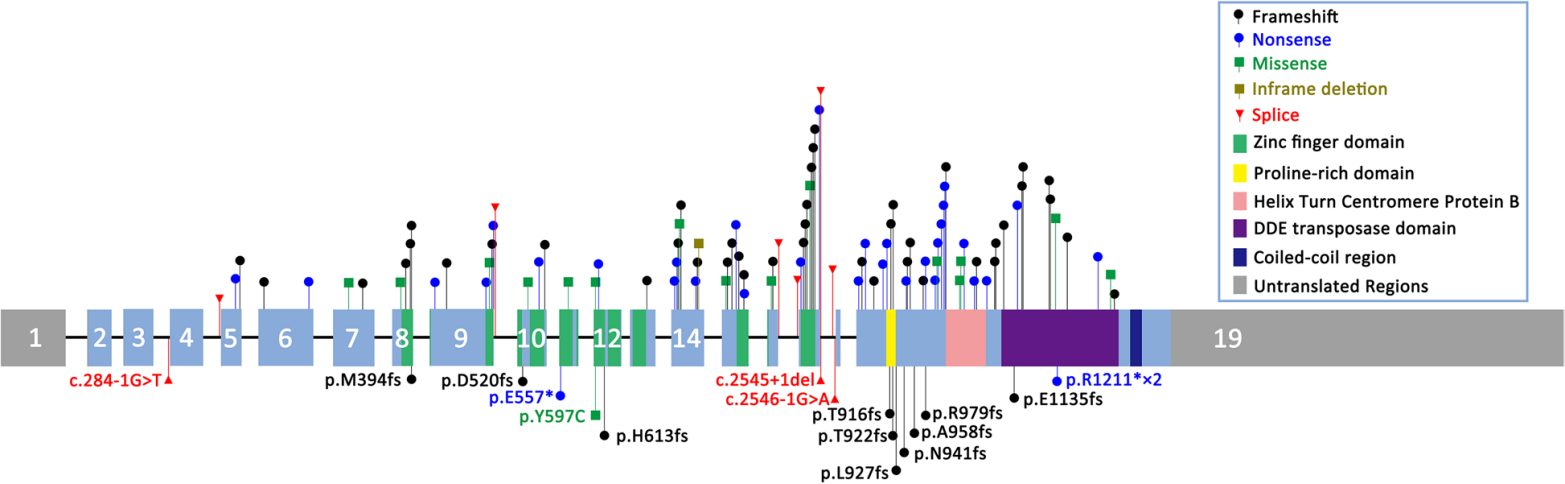
Schematic representation of reported variants in the POGZ gene. Variants in cases with congenital heart disease are shown below the exon structure, and variants in other cases are shown above

Peer-reviewed articles were identified by searching PubMed with the search terms: “*POGZ*” and “White-Sutton syndrome.” A total of 141 cases of White-Sutton syndrome caused by mutation of *POGZ* were identified [5–19, 22–36] (Supplementary Table 1, Fig. 2). The types of mutations in these cases included frameshift mutation (61/141, 43.3%), nonsense mutation (49/141, 34.8%), splicing mutation (9/141, 5.6%), large deletion (3/141, 2.5%), missense mutation (17/141, 12.1%), intronic mutation (1/141, 0.7%) and in-frame deletion (1/141, 0.7%). Overall, 80.1% of the reported mutations were null variants, which suggests that loss of function is the main mechanism of pathogenicity. A previous function study revealed that de novo mutations Q1042R and R1008X in *POGZ* disrupt its DNA-binding activity, and a de novo missense mutation (Q1042R) is associated with an approximately 60% reduction in the DNA-binding activity of *POGZ* [37], which further proves that loss of function is the pathogenic mechanism. The mutation identified in the present case is a novel frameshift mutation, which is a common type of loss-of-function mutation.

The clinical descriptions of the 141 cases included varying phenotypic details, and a relatively detailed phenotype information was provided for 125 cases. Among those 125 cases, 16 cases (16/125, 12.5%) had previously received a diagnosis of congenital heart disease [5, 11, 14, 17, 19, 22, 24, 25, 27, 29, 31, 36]. In addition, we found in the Decipher database (https://decipher.sanger.ac.uk/) two cases (Patients: 333,151 and 284,226) with heart disease and a pathogenic mutation in the *POGZ* gene to which the patients’ whole phenotype was attributed. Therefore, we found a total of 19 cases (including the present case) with congenital heart disease (Tables 1 and 2). Among these 19 cases with a cardiovascular defect, clinical exome sequencing was performed for 6 cases, Trio-WES for 4 cases, both Trio-WGS and microarray analysis for 1 case, and both Trio-WES and microarray analysis for 4 cases. As such, the patients’ genetic test results were relatively comprehensive. However, no other suspected pathogenic mutations were reported in these cases. In particular, four of the cases were reported in studies on congenital heart disease [11, 19, 22], and no other disease-causing mutations were found in genes associated with congenital heart disease. In conclusion, we believe the likelihood of other another underlying genetic etiology causing congenital heart disease in these patients with White-Sutton syndrome is low. In addition, according to the cases we reviewed, the incidence of congenital heart disease in patients with POGZ mutation was 12.5%, compared with only 0.8 ~ 1% in all newborns [38, 39]. This finding suggests that the incidence of congenital heart disease is significantly higher in patients with POGZ mutation than in the general population and supports the hypothesis that congenital heart disease is a relatively uncommon feature in White-Sutton syndrome.

**Table 1:** Characteristics of White-Sutton cases with congenital heart disease

Individual	Our case	157 Reuter et al. (2020) [11]	1-00961 Homsy et al. (2015) [22]	1-02312^a^ Homsy et al. (2015) [22]	1-07689 Jin et al. (2017) [19]	PT23 Assia Batzir et al. (2020）[5]	3 cases Assia Batzir et al. (2020)	Patient:333,151 Decipher database	Patient:284,226 Decipher database
**Gender**	F	NA	NA	NA	NA	M	NA	NA	NA
**Age at onset**	Birth	NA	NA	NA	NA	2 years	NA	NA	NA
**Mutation(s)**	c.2746del p.Thr916Profs*12	c.3403del p.Glu1135Argfs*3	c.284-1G > T	c.1838 A > G p.His613Arg	c.1558_1559delinsT p.Asp520Phefs*7	c.1669G > T p.Glu557*	NA	c.1837​del p.His613Metfs*13	c.2935 C​>T p.(Arg979*)
**Genetic testing**	Trio-WES, prenatal karyotype, microarry	Trio-WGS, prenatal microarray, FISH 22q	Trio-WES	Trio-WES	Trio-WES	Clinical exome sequencing	Clinical exome sequencing		
**Inheritance**	De novo	De novo	De novo	De novo	De novo	De novo	NA	De novo	De novo
**Cardiovascular defect**	Atrial septal defect (18 × 23 × 22 mm)	Mitral atresia, aortic atresia	Hypoplastic right ventricle; pulmonary atresia congenital; pulmonary atresia, intact ventricular septum	Aortic arch hypoplasia; atrial septal defect, secundum; hypoplastic aortic annulus; hypoplastic left ventricle; mitral stenosis, valvar; ventricular septal defect, single	Congenital coronary anomaly; DORV, ventricular defect committed to aorta; left aortic arch with normal branching pattern; SDD; subaortic conus; ventricular septal defect, malalignment	Mitral valve prolapse	Atrial septal defect/ patent foramen ovale (2 patients), and aortic root dilatation (1 patient)	Abnormality of the cardiovascular system	Dextrocardia
**Developmental delay**	YES	YES	YES	NO	NA	YES	NA	NA	NA
**Seizure**	YES	NA	NA	NO	NA	NO	NA	NA	NA
**ASD**	NA	NA	NO	NO	NA	NO	NA	NA	NA
**Microcephaly**	YES	NA	NA	NO	NA	NO	NA	NA	NA
**Gastrointestinal issues**	YES	YES	NA	NO	NA	NA	NA	NA	NA
**Hearing impairment**	YES	NA	NA	NO	NA	NA	NA	NA	NA
**Abnormal brain imaging**	YES	NA	NA	NO	NA	NO	NA	NA	NA
**Other**	Recurrent respiratory infection	Borderline short stature	Laryngo bronchio tracheomalacia, subglottic cyst and learning disability	NA	NA	Myopia, astigmatism, pectus excavatum arachnodactyly	NA	Nervous system abnormality	Aganglionic megacolon

^a^Pathogenicity of the variant is dubious

**Table 2:** Characteristics of White-Sutton cases with congenital heart disease

Individual	Individual 10 Murch et al. (2022) [25]	L01 Nagy,Dóra et al. (2022) [24]	Patient 2 White et al. (2016) [17]	Patient 1 Dentici et al. (2017) [14]	Patient Pascolini et al. (2020) [36]	Patient Trimarchi et al. (2021) [31]	Patient 8 Garde et al. (2021) [27]	Patient Dal et al. (2021) [29]
**Gender**	male	male	Female	Female	Male	Female	Male	Male
**Age at onset**	Birth	NA	NA	Birth	NA	Birth	NA	NA
**Mutation**	c2933_2934dupTT p.Arg979Phefs*3	c.2873_2874delCA; p.Ala958Valfs*6	c.2763dupC p.Thr922Hisfs*22	c.2820dupG p.Asn941Glufs*3	c.3631 C > T p.Arg1211*	c,2546-1G > A	c.2545 + 1delG	c.3631 C > T p.Arg1211*
**Genetic testing**	Trio-WES, karyotype, array	NA	diagnostic WES	WES, chromosomal microarray	Family-based WES	Trio-WES, array-CGH	NA	Exome sequencing
**Inheritance**	De novo	De novo	De novo	De novo	De novo	De novo	De novo	De novo
**Cardiovascular defect**	Atrial septal defect	Atrial septal defect	Atrial septal defect, Patent ductus arteriosus, patent foramen ovale	Atrial septal defect	Aortic bicuspid valve with mild ascending aorta dilatation	Congenital heart disease	Atrial septal defect	Dextrocardia
**Developmental Delay**	YES	YES	YES	YES	YES	YES	YES	YES
**Seizure**	NO	YES	NO	YES	NO	NO	NO	NA
**ASD**	NA	NA	NO	YES	YES	NA	NA	NA
**Microcephaly**	YES	YES	YES	YES	NO	YES	NA	NA
**Gastrointestinal issues**	YES	YES	YES	YES	Yes	YES	NA	NA
**Hearing impairment**	Yes	NO	Yes	Yes	Yes	YES	Yes	NA
**Abnormal brain imaging**	NA	NA	YES	YES	YES	YES	YES	NA
**Other**	Prominent right eye with strabismus, cystic hygroma, pyloric stenosis, bilateral cryptorchidism	Pendle nystagmus and choroidal atrophy	Congenital diaphragmatic hernia; duplicated renal collecting system, cortical blindness	Vitiligo	Overweight	Dystonia	Common mesentary, micropenis, cryptorchidism, vision impairment	Overweight

All of the variants in cases with congenital heart disease were truncation variants (i.e., frameshift, nonsense, splicing and large deletion mutation) except for c.1838 A > G (p.His613Arg). The only missense mutation, c.1838 A > G reported by Homsy et al. [19], was identified de novo in a case without neurodevelopmental disabilities. According to the Sequence Variant Interpretation Working Group (SVI WG) general recommendations for using ACMG/AMP criteria (https://clinicalgenome.org/working-groups/sequence-variant-interpretation/), c.1838 A > G was reclassified as a variant of uncertain significance, and this patient lacked other pathogenomic features (specifically neurodevelopmental disabilities) of White-Sutton syndrome. Thus, we believe that the pathogenicity of c.1838 A > G is dubious, and more evidence is needed to support it. Therefore, we only discuss the remaining 18 cases when considering the relationship between congenital heart disease and White-Sutton syndrome. The variants in these cases were scattered across genes and not concentrated in specific domains (Fig. 2). Moreover, two mutations, c.2545 + 1del and c.1180_1181del, have been reported in patients with and without congenital heart disease. Therefore, no significant difference was found in the type or distribution of variants between patients with and without congenital heart disease. In terms of the type of cardiac abnormalities, two of these 18 cases had no detailed phenotype of congenital heart disease. The cardiac abnormalities in the remaining 16 cases varied widely and included many types of congenital heart disease (Table 1). It is worth noting that atrial septal defects were presented in 8 cases (8/16, 50%) (including the present case), making this the most common defect type.

Animal models are an important tool for understanding the relationship between genes and disease. A mouse model with a heterozygous or homozygous nervous system-specific deletion of the *Pogz* gene mimicked several of the human symptoms, showing microcephaly, growth impairment, increased sociability, and learning and motor deficits [40]. Mice heterozygous for the Q1038R mutation exhibited decreased brain size, decreased cortical thickness, and ASD-related behavioral abnormalities [4]. Significantly, Complete knockout of *Pogz* [41] or homozygosity for the Q1038R mutation in mice [4] both cause early embryonic lethality. Micro computed tomography (CT) scanning of Q1038R homozygous mouse embryos (E15.5) showed a ventricular septal defect, which was suspected to result in embryonic lethality. This finding in a mouse model further supports the relationship between congenital heart disease and *POGZ* mutation.

In summary, we herein described a new White-Sutton syndrome patient with a novel frameshift de novo *POGZ* variant, c.2746delA (p.Thr916ProfsTer12). Furthermore, we reviewed all previously reported cases of White-Sutton syndrome with *POGZ* mutation and focused on patients with congenital heart disease. Our findings suggest that the White-Sutton syndrome phenotype may align with congenital heart disease. More cases showing a similar presentation would support our findings. In addition, the role of *POGZ* in cardiac development has not been functionally verified, and such analysis may be needed in the future.

## Supplementary Information

**Additional file 1.** 

## Data Availability

The datasets for this article are not publicly available due to concerns regarding participant/patient anonymity. Requests to access the datasets should be directed to the corresponding author.

## Abbreviations

WES: Whole-exome sequencing
